# Intrathoracic gossypiboma presenting 47 years later as a purulent fistula: a case report

**DOI:** 10.1186/s40792-022-01479-6

**Published:** 2022-06-24

**Authors:** Shahab Rafieian, Matin Vahedi, Javad Sarbazzadeh, Hesam Amini, Reza Ershadi

**Affiliations:** Department of Thoracic Surgery, Imam Khomeini Hospital Complex, Tehran University of Medical Sciences, Qarib Street, Tehran, Iran

**Keywords:** Video-assisted thoracoscopic surgery, Gossypiboma, Purulent fistula

## Abstract

**Background:**

Intrathoracic gossypiboma is a consequence of retained sponge/swap, gauzoma, muslinoma, textiloma, or cottonoid in the thoracic cavity during surgery. The thoracic cavity is of the rarest place for gossypiboma as these entities most occur after abdominal surgery.

**Case presentation:**

We report a case of intrathoracic gossypiboma that was missed for an extended period of time with no symptoms and was successfully treated with surgical intervention.

**Conclusions:**

The rarity of gossypiboma necessitates a high index of suspicion for correct diagnosis. Gossypiboma is often difficult to diagnose, leading to misdiagnosis and unnecessary interventions. It is important to consider this entity as a diagnosis in any case with an unexplained or unusual presentation during the postoperative period.

## Introduction

Several factors contribute to patient morbidity and mortality during surgery. Retained sponge/swap, gauzoma, muslinoma, textiloma, or cottonoid are examples of these, and they all lead to gossypiboma [1, 2]. Gossypiboma can occur in any cavity, although the abdomen, pelvis, and thorax are the most common [1]. In unresolved situations, they frequently lead to major complications that result in rehospitalization, reoperation, and even death. In the case of an intrapleural opacity following surgery, a differential diagnosis of gossypiboma should always be explored. A thorough clinical history, such as the onset of mass development, can provide a clue to its origin, which can then assist the treatment [3]. The gossypiboma rate of occurrence varies between 1 in 1000 and 1 in 10,000 cases. Nonetheless, determining the exact incidence of gossypiboma is difficult due to widespread underreporting due to the risk of medicolegal repercussions [4]. In general, thoracic gossypiboma is not a common diagnosis. A recent analysis found only 40 cases in the English literature [1]. Clinically, patients may be asymptomatic for a long period or present with discomfort, nausea, vomiting, or a palpable lump, which can take anywhere from a few hours to years to diagnose [2, 3]. Here, we report a case of intrathoracic gossypiboma that was missed for a long period of time with no symptoms.

## Case presentation

A 62-year-old male patient was admitted to our department with a purulent fistula at the site of previous surgery in the right posterolateral thoracic area. The fistula was painful, warm, and erythematous with induration and continuous milky purulent drainage. He had a past medical history of diabetes mellitus from 20 years ago, which was treated with insulin injections. He also had two previous surgeries, the first was right posterolateral thoracotomy and pneumonectomy for the management of chronic tuberculosis about 47 years ago, and the other one was abdomino-pelvic resection for the management of colorectal cancer. The patient had developed a purulent lesion from two years ago, but mentioned no other symptoms. He also mentioned that his diabetes mellitus was severe in these 2 years. On physical examination, he had a 3*3 cm lesion with purulent secretion in the right posterolateral thoracic area. The surrounding tissue was red and tender.

**Fig. 1:**
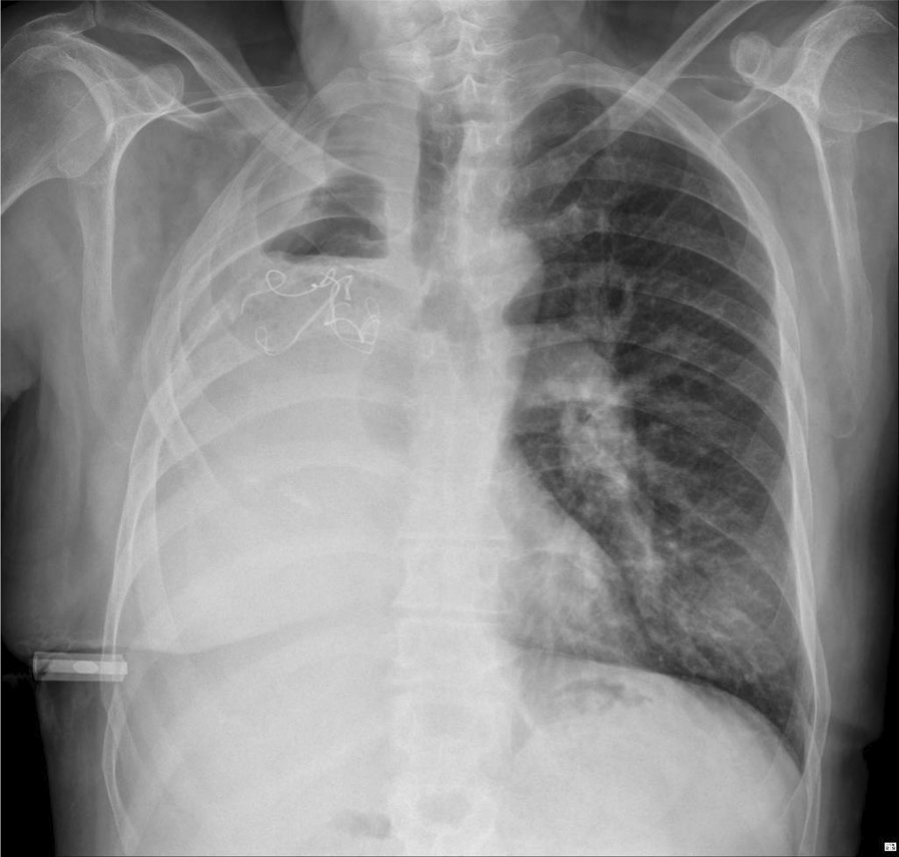
Chest X-ray showing right hemithoracic opacity with thread-like calcifications

**Fig. 2:**
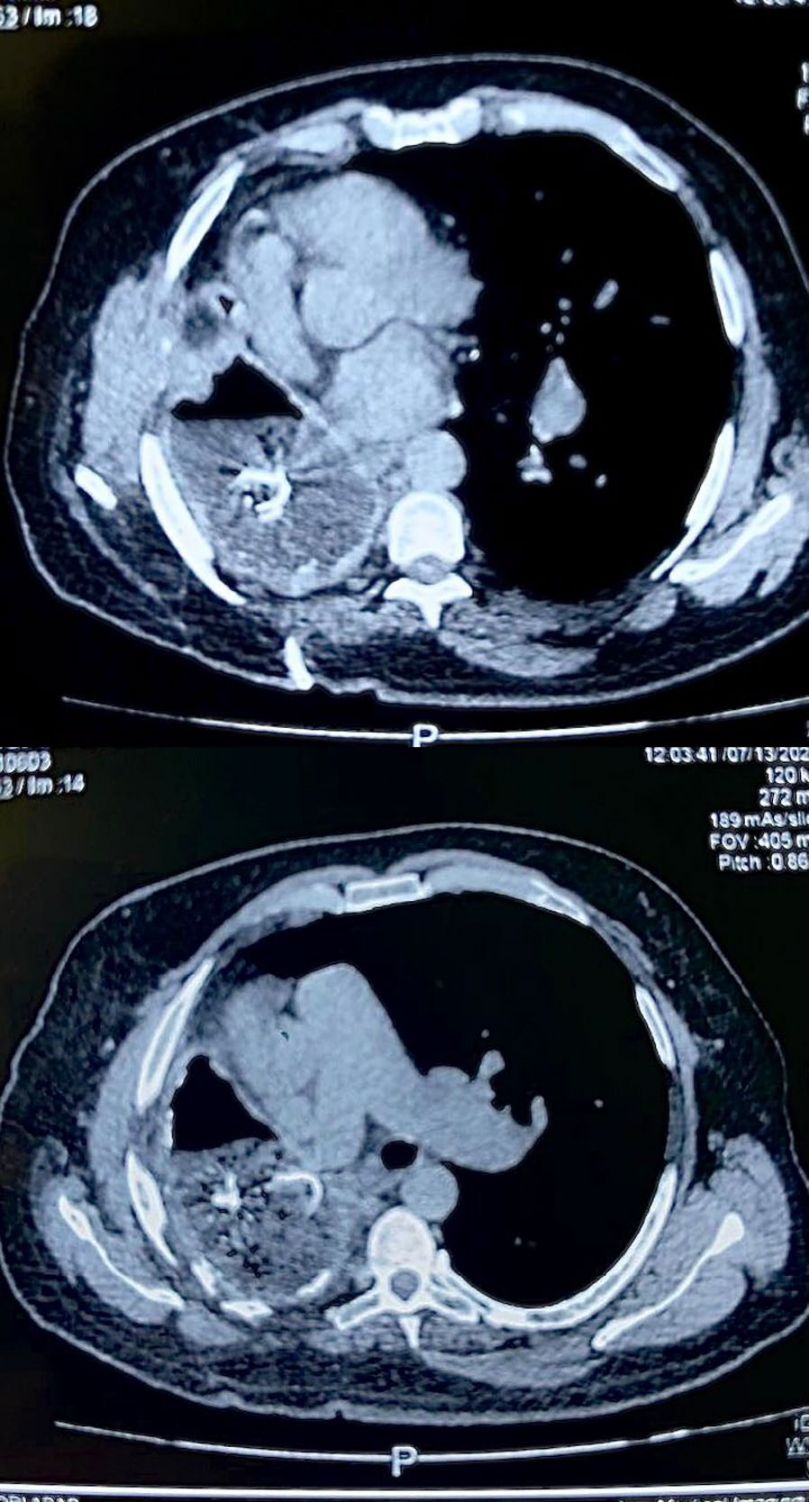
Axial thoracic CT scan showing gossypiboma in occupying right hemithoracic cavity

**Fig. 3:**
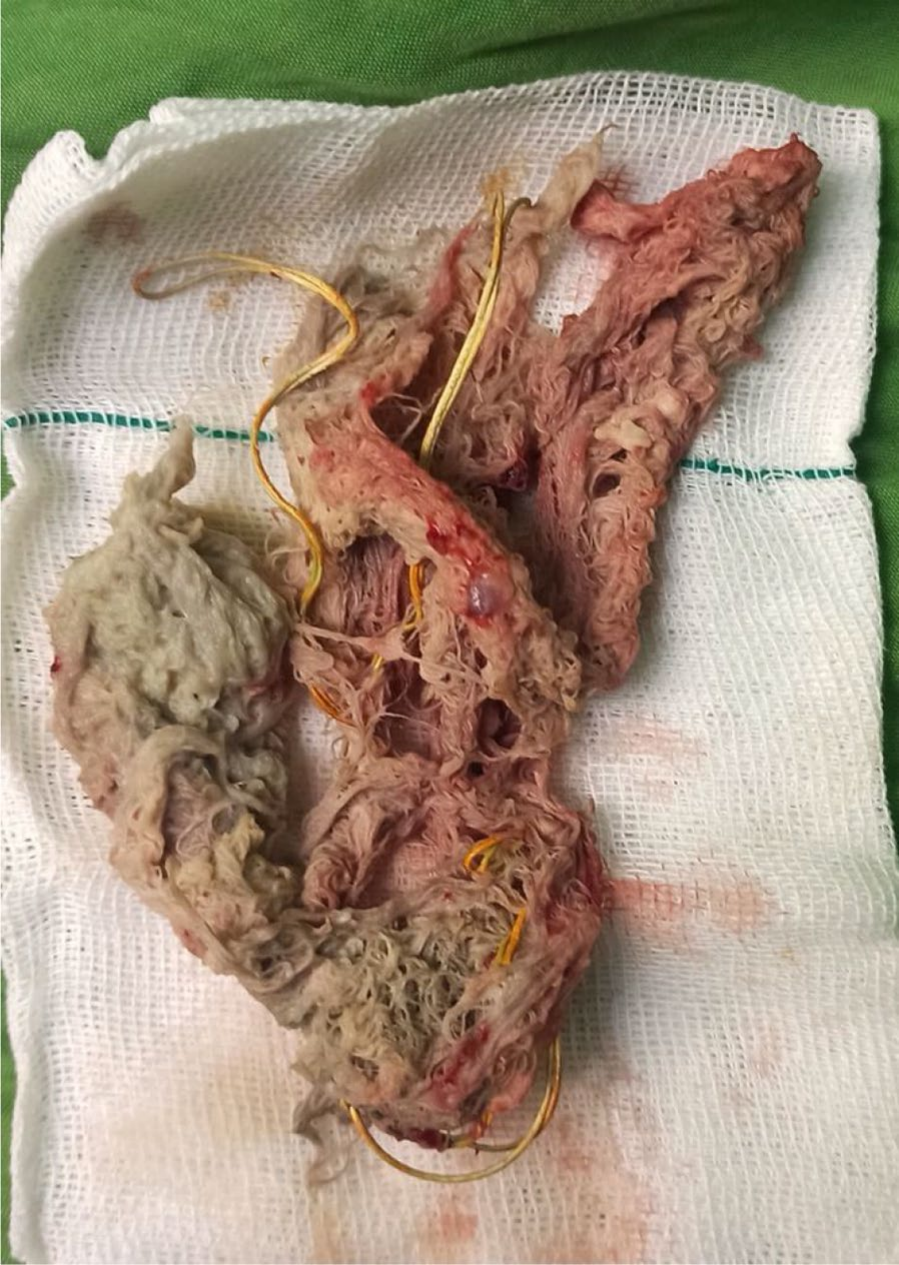
Gross image of the removed gossypiboma

Chest X-ray showed opacities covering the whole right hemithorax and radiopaque strip (Fig. 1). The patient was investigated with a chest computed tomography (CT) scan without contrast, which showed a giant lesion within the right thoracic cavity with thread-like calcifications (Fig. 2). With suspicion of gossypiboma, right video-assisted thoracoscopic surgery was planned. Preoperative lab testing showed a white blood cell count of 10,600/μL with 81.6% polymorphonuclear neutrophils and 7.9% lymphocytes. C-reactive protein was in the upper limit of normal. Other blood examinations were normal. Microbial examination of the lesion showed infection with E. coli, which was resistant to ceftriaxone and ampicillin sulbactam. An infected surgical sponge was detected in the surgery. Due to severe adhesion to thoracic structures and mediastinum, we had to convert the operation to a right posterolateral thoracotomy. And it was removed from the thoracic cavity without any complications (Fig. 3).

Along with the sponge, a considerable amount of blood and suppuration was also removed from the thoracic cavity. A chest tube was inserted to drain the remnant purulent material. We used a chest tube and feeding tube catheter to irrigate the pleural space with warm normal saline (1000 cc) three times/day. The postoperative irrigation continued until the chest tube fluid became clear, lab examination became normal, and the patient did not have any signs of fever and infection. The chest tube was removed 3 days after surgery. The patient was then discharged with no symptoms or complications.

## Discussion

Gossypiboma is a reaction to a foreign body within body cavities. It results from a reaction against the cotton matrix in a sponge and then the formation of a mass [5]. Typically, a retained sponge induces an abscess due to granulation and exudative reaction, but does not activate any biochemical reaction. Notwithstanding, the risk of bacterial superinfection and creation of fistulas increases with time. The first case of gossypiboma was described in 1884 as ‘a cotton sponge left in a patient’s abdomen’ [5]. In a recent study, Gulsen et al. [5] reviewed the cases of intrathoracic gossypiboma. The time from the surgery to diagnosis ranged from 3 weeks to 52 years [1, 5]. Our case was diagnosed approximately 47 years after the initial surgery. Most patients with intrathoracic gossypiboma are symptomatic, presenting with chronic cough, shortness of breath, chest pain, fever and weight loss. There are also a few cases of asymptomatic gossypiboma [5]. Typical presentation and typical radiological findings narrowed our differential diagnosis. However, this is not the case in all cases of intrathoracic gossypiboma. Gossypiboma can be misdiagnosed as hematoma [6], bronchiectasis [7], malignancy [8], aspergilloma [9], hydatid cyst [10], and empyema [5]. The most frequent location of gossypibomas is the abdomen. Nonetheless, they could be less frequently found in the pelvis or thorax, as in our case [11]. Obesity, emergency surgeries, and improper counting of materials at the end of procedures are associated with an increased risk of gossypibomas [12]. However, none of these were present in our case.

Radiological examination, including X-ray and CT scan, are the main route of diagnosis. Well-circumscribed soft tissue density or calcification can be observed in chest X-rays. However, a CT scan is the preferred option [13]. Abdominal and intrathoracic gossypibomas are characterized by spongiform patterns that could be misdiagnosed as empyema or abscess [14].

The World Health Organization's (WHO) global challenge 'Safe Surgery Saves Lives' in 2007 drew on literature and clinical experience from around the world to produce a core set of surgical safety standards. The WHO's Surgical Safety Checklist was created to improve surgical care safety, and it has resulted in a significant decrease in both morbidity and mortality since its implementation. Nonetheless, in a previously operated patient, gossypiboma should be considered if no other differential diagnosis or source of infection can be identified. This is true even when there is no radiopaque line present, as in the cases reported by Dubois et al. [3] and Bakan et al. [15], where gossypiboma was discovered despite the sponge lacking a radiopaque line. Finally, it is essential to note that a history of previous thoracic surgery is not the only prerequisite for intrathoracic gossypiboma, as it can also arise from abdominal or spinal surgery, making those cases unique and worthy of discussion [15].

## Conclusion

We presented a case of intrathoracic gossypiboma that had been asymptomatic for a long period of time. The rarity of gossypiboma necessitates a high index of suspicion for correct diagnosis. Surgical intervention is the primary treatment of gossypiboma.

## Abbreviations

CT: Computed tomography
WHO: World Health Organization
